# Supplementary material for: KLF3 and PAX6 are candidate driver genes in late-stage, MSI-hypermutated endometrioid endometrial carcinomas
Source: PLoS One. 2022 Jan 26;17(1):e0251286. doi: 10.1371/journal.pone.0251286 (PMC8791453; doi:10.1371/journal.pone.0251286)
Supplement: S1 Fig — (PPTX) [file pone.0251286.s001.pptx]

## Slide 1
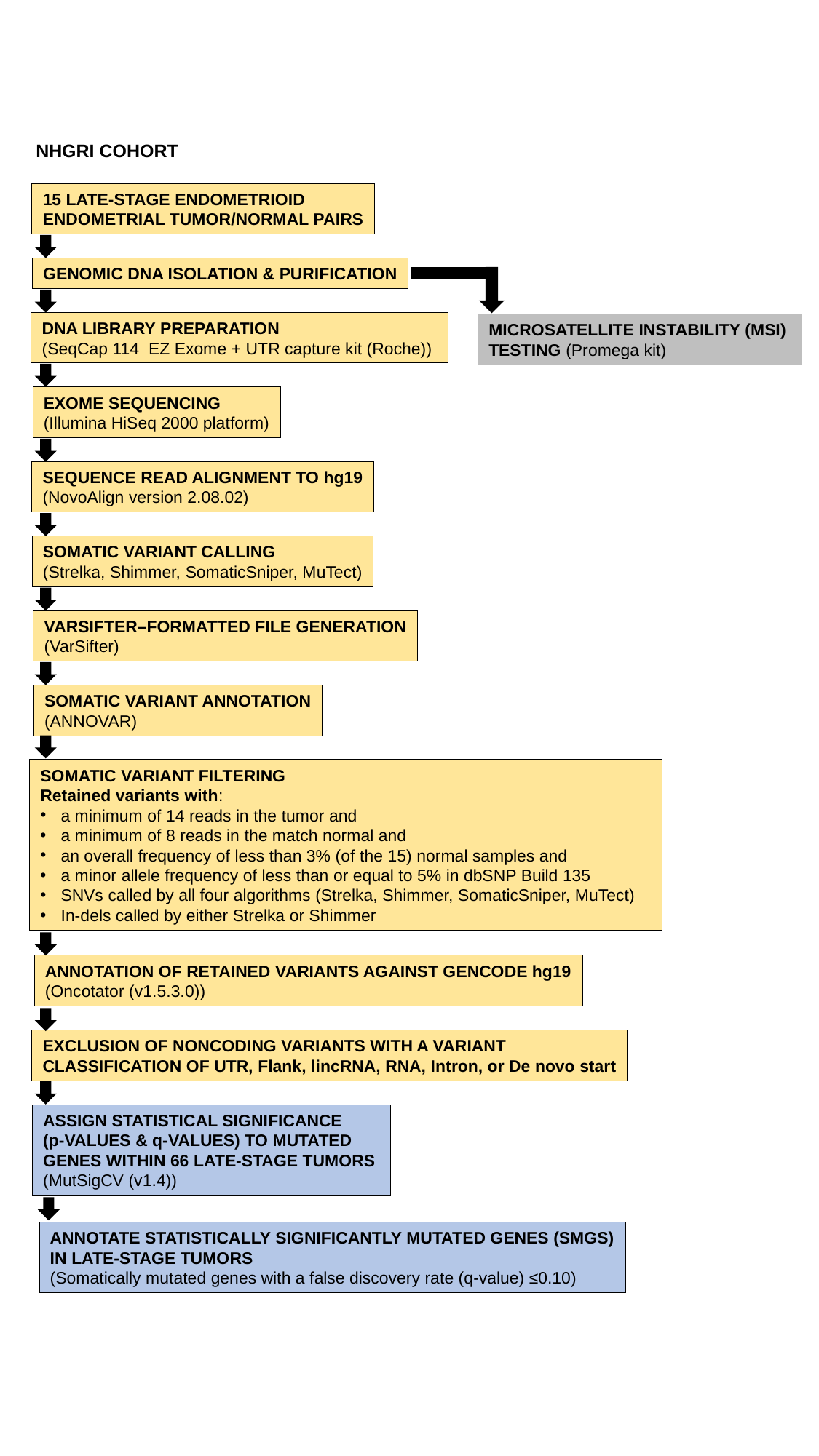

NHGRI COHORT
15 LATE-STAGE ENDOMETRIOID
ENDOMETRIAL TUMOR/NORMAL PAIRS
GENOMIC DNA ISOLATION & PURIFICATION
DNA LIBRARY PREPARATION
(SeqCap 114  EZ Exome + UTR capture kit (Roche))
MICROSATELLITE INSTABILITY (MSI)
TESTING (Promega kit)
EXOME SEQUENCING
(Illumina HiSeq 2000 platform)
SEQUENCE READ ALIGNMENT TO hg19
(NovoAlign version 2.08.02)
SOMATIC VARIANT CALLING
(Strelka, Shimmer, SomaticSniper, MuTect)
VARSIFTER–FORMATTED FILE GENERATION
(VarSifter)
SOMATIC VARIANT ANNOTATION
(ANNOVAR)
SOMATIC VARIANT FILTERING
Retained variants with:
a minimum of 14 reads in the tumor and
a minimum of 8 reads in the match normal and
an overall frequency of less than 3% (of the 15) normal samples and
a minor allele frequency of less than or equal to 5% in dbSNP Build 135
SNVs called by all four algorithms (Strelka, Shimmer, SomaticSniper, MuTect)
In-dels called by either Strelka or Shimmer
ANNOTATION OF RETAINED VARIANTS AGAINST GENCODE hg19
(Oncotator (v1.5.3.0))
EXCLUSION OF NONCODING VARIANTS WITH A VARIANT
CLASSIFICATION OF UTR, Flank, lincRNA, RNA, Intron, or De novo start
ASSIGN STATISTICAL SIGNIFICANCE
(p-VALUES & q-VALUES) TO MUTATED
GENES WITHIN 66 LATE-STAGE TUMORS
(MutSigCV (v1.4))
ANNOTATE STATISTICALLY SIGNIFICANTLY MUTATED GENES (SMGS)
IN LATE-STAGE TUMORS
(Somatically mutated genes with a false discovery rate (q-value) ≤0.10)
