# Supplementary material for: KLF3 and PAX6 are candidate driver genes in late-stage, MSI-hypermutated endometrioid endometrial carcinomas
Source: PLoS One. 2022 Jan 26;17(1):e0251286. doi: 10.1371/journal.pone.0251286 (PMC8791453; doi:10.1371/journal.pone.0251286)
Supplement: S2 Fig — (PPTX) [file pone.0251286.s002.pptx]

## Slide 1
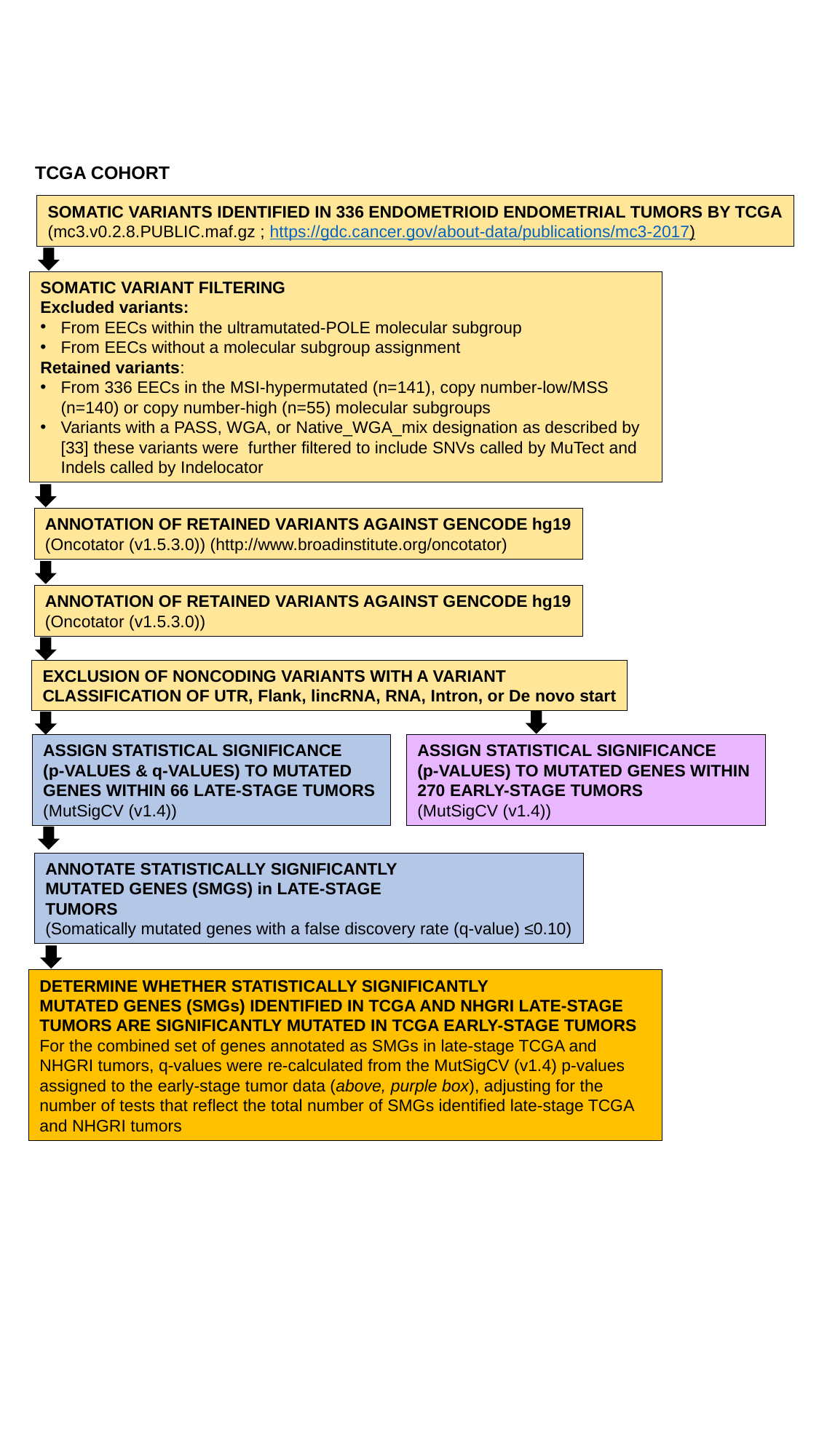

TCGA COHORT
SOMATIC VARIANTS IDENTIFIED IN 336 ENDOMETRIOID ENDOMETRIAL TUMORS BY TCGA
(mc3.v0.2.8.PUBLIC.maf.gz ; https://gdc.cancer.gov/about-data/publications/mc3-2017)
SOMATIC VARIANT FILTERING
Excluded variants:
From EECs within the ultramutated-POLE molecular subgroup
From EECs without a molecular subgroup assignment
Retained variants:
From 336 EECs in the MSI-hypermutated (n=141), copy number-low/MSS (n=140) or copy number-high (n=55) molecular subgroups
Variants with a PASS, WGA, or Native_WGA_mix designation as described by [33] these variants were further filtered to include SNVs called by MuTect and Indels called by Indelocator
ANNOTATION OF RETAINED VARIANTS AGAINST GENCODE hg19
(Oncotator (v1.5.3.0)) (http://www.broadinstitute.org/oncotator)
ANNOTATION OF RETAINED VARIANTS AGAINST GENCODE hg19
(Oncotator (v1.5.3.0))
EXCLUSION OF NONCODING VARIANTS WITH A VARIANT
CLASSIFICATION OF UTR, Flank, lincRNA, RNA, Intron, or De novo start
ASSIGN STATISTICAL SIGNIFICANCE
(p-VALUES & q-VALUES) TO MUTATED
GENES WITHIN 66 LATE-STAGE TUMORS
(MutSigCV (v1.4))
ASSIGN STATISTICAL SIGNIFICANCE
(p-VALUES) TO MUTATED GENES WITHIN
270 EARLY-STAGE TUMORS
(MutSigCV (v1.4))
ANNOTATE STATISTICALLY SIGNIFICANTLY
MUTATED GENES (SMGS) in LATE-STAGE
TUMORS
(Somatically mutated genes with a false discovery rate (q-value) ≤0.10)
DETERMINE WHETHER STATISTICALLY SIGNIFICANTLY
MUTATED GENES (SMGs) IDENTIFIED IN TCGA AND NHGRI LATE-STAGE
TUMORS ARE SIGNIFICANTLY MUTATED IN TCGA EARLY-STAGE TUMORS
For the combined set of genes annotated as SMGs in late-stage TCGA and NHGRI tumors, q-values were re-calculated from the MutSigCV (v1.4) p-values assigned to the early-stage tumor data (above, purple box), adjusting for the number of tests that reflect the total number of SMGs identified late-stage TCGA and NHGRI tumors
