# Supplementary figures and images for: KLF3 and PAX6 are candidate driver genes in late-stage, MSI-hypermutated endometrioid endometrial carcinomas
Source: PLoS One. 2022 Jan 26;17(1):e0251286. doi: 10.1371/journal.pone.0251286 (PMC8791453; doi:10.1371/journal.pone.0251286)

## Slide 1
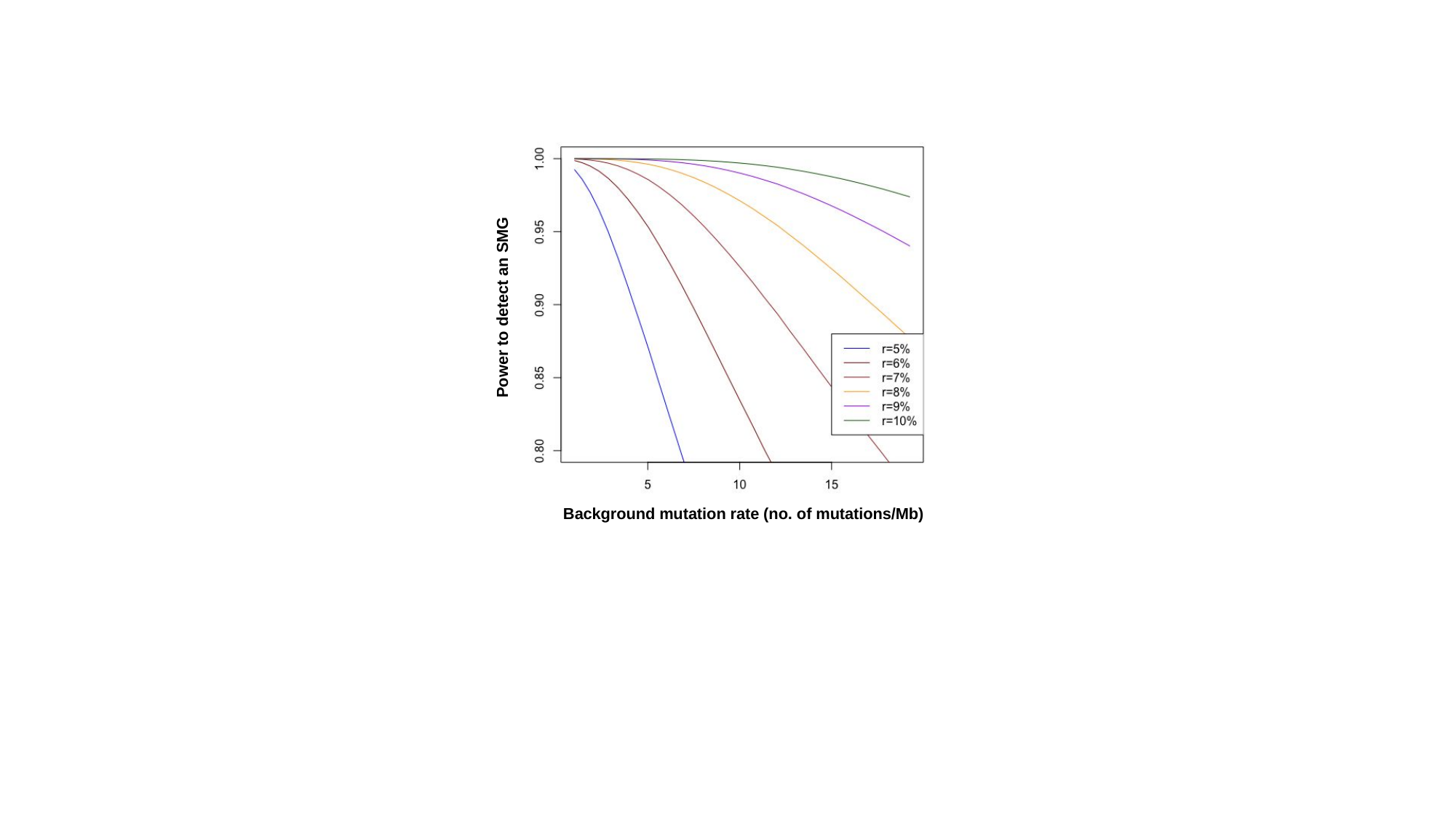

Power to detect an SMG
Background mutation rate (no. of mutations/Mb)

Supplement: S3 Fig — Curves show statistical power for different percentages (r) of tumors that are somatically mutated. Calculations were performed as described in the text, assuming 270 tumors and 14 gene tests completed. (PPTX) [file pone.0251286.s003.pptx]

## Slide 1
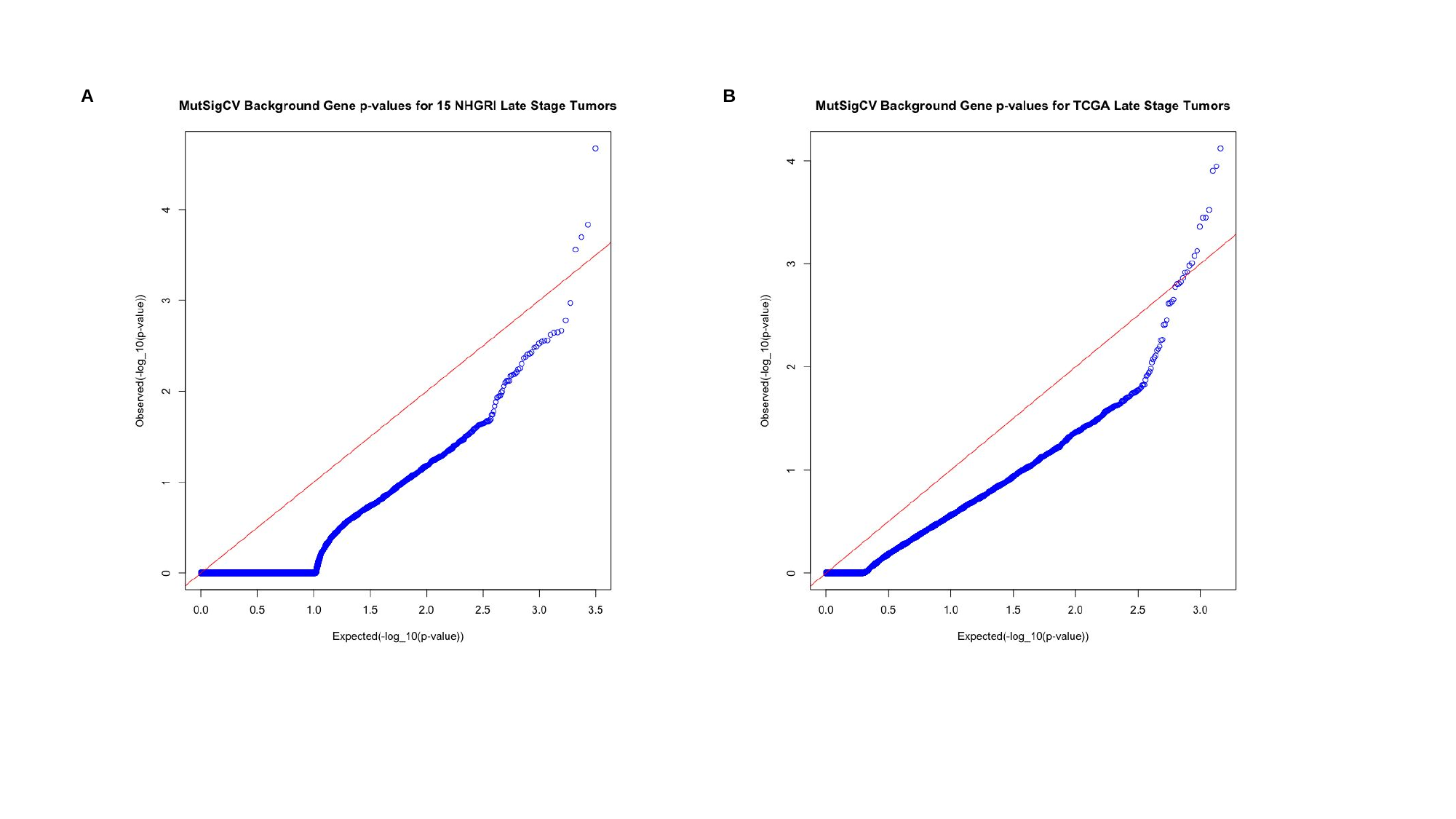

B
A

Supplement: S4 Fig — Q-Q plots for MutSigCV’s p-values for differing mutation rates in background genes in (A) the 15 late-stage tumors sequenced and analyzed at NHGRI and (B) the set of 66 late-stage tumors from the TCGA project. Deviation from uniform p-value distribution here is a result of MutSigCV’s assigned p-value and probably due to the limited number of tumors analyzed. (PPTX) [file pone.0251286.s004.pptx]
